# Genotypic Characterization of Human Parvovirus B19 Circulating in the 2024 Outbreak in Tuscany, Italy

**DOI:** 10.3390/pathogens14020121

**Published:** 2025-01-28

**Authors:** Giada Beligni, Giulia Alessandri, Maria Grazia Cusi

**Affiliations:** 1 Virology Unit, Department of Medical Biotechnologies, University of Siena, 53100 Siena, Italy; giada.beligni@unisi.it (G.B.); giulia.alessandri@student.unisi.it (G.A.); 2 Microbiology and Virology Unit, Santa Maria delle Scotte University Hospital, Viale Bracci 1, 53100 Siena, Italy

**Keywords:** Parvovirus B19, outbreak, G1a genotype

## Abstract

Human Parvovirus B19 (hB19V) is a widespread virus, causing *erythema infectiosum* in children and several clinical manifestations from acute to persistent infections in adults. In early 2024, ECDC reported an increased human Parvovirus B19 circulation in 14 European countries. A hB19V outbreak was also reported in Tuscany, Italy, prompting a detailed investigation of its genetic characteristics. In this context, through strict monitoring of circulating strains via next-generation sequencing (NGS), we carried out a phylogenetic analysis based on the whole of hB19V genomes. Phylogenetic clustering assigned all isolates to the G1a genotype, although with some mutations in NS1, VP1, and VP2, compared to the reference strains. Further characterization of these variants is necessary to fully assess their potential implications for public health. This study provides valuable insights into the spread of Parvovirus B19 and underlines the importance of continuous genomic surveillance to monitor and respond to possible hB19V epidemics that could impact public health.

## 1. Introduction

Human parvovirus B19 (hB19V) is a single-stranded DNA virus (ssDNA) belonging to the Parvoviridae family. It is the aetiologic agent of the *erythema infectiosum* (fifth disease) in children and it can infect adults, particularly those who are immunosuppressed or have chronic hematological diseases, causing a wide range of clinical manifestations [1]. Although most of the patients are asymptomatic or have mild symptoms, when hB19V affects adults, it can lead to more serious conditions, such as arthropathy, aplastic crises, and hydrops fetalis in pregnant women [2,3]. HB19V can be transmitted via respiratory secretions, hand-to-mouth contact, blood transfusion, or transplacental transmission [4]. The incubation period varies from 4 to 14 days after exposure, but it can last up to 3 weeks [5]. HB19V infections usually follow a seasonal pattern, with incidence peaks typically occurring in late spring or early summer, especially in temperate countries [6]. Larger epidemics have been reported every 3–4 years, with fluctuation in the number of cases across different seasons [7]. Since the first few months of the 2024, and especially during late spring-early summer, an unexpected surge in hB19V was noted in a lot of European countries [8,9], including Italy. Indeed, a recent study reported an outbreak of hB19V in pregnant women in Northern Italy [10]. In Tuscany, specifically in Siena area, only three cases were recorded in our laboratory in 2023; a lot of requests arrived at the Virology Unit and various samples were positively diagnosed from January to September 2024.

PCR-based molecular assay is widely employed for hB19V diagnosis; however, many routine clinical laboratories rely solely on serology, using commercial ELISA or immunofluorescence-based assays for anti hB19V IgG and IgM antibodies analysis [11].

**Figure 1:**
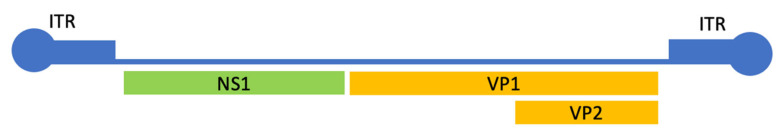
A representation of the hB19V genome. The 5596nt genome is composed of 5′ and 3′ inverted terminal repeats, and three main genes: the non-structural protein1 (NS1), the viral protein 1 (VP1), and the viral protein 2 (VP2). The two proteins share the whole sequence of VP2, but VP1 has an additional N-terminal domain known as the VP1 unique region (VP1u).

HB19V genome is a 5596-nucleotide-long ssDNA molecule, encoding for several structural and non-structural proteins. Its genome is composed of two main regions (Figure 1): the non-coding region, formed by two inverted terminal repeats (ITR) and mainly involved in viral replication and the encapsidation process, and the coding region for four non-structural proteins (NS1, 7.5-, 9-, and 11-kDa) and two structural proteins (VP1 and VP2).

ITRs are 383 nucleotides in length and form imperfect palindromic sequences that can be folded into hairpins expressed in two alternative orientations. They play a crucial role in viral replication processes. Indeed, the ability of palindromic sequences to fold in self-priming, hairpin secondary structures, and the presence of specific cis-recognition sequences acting as origins of replication, allow replication of the viral DNA through a rolling hairpin mechanism [12].

The non-structural 1 protein (NS1) is involved in viral replication, cytotoxicity, and host immune response. The structural VP proteins include VP1, containing a unique N-terminus (VP1u), and are critical for virus entry via the phospholipase A2 (vPLA2) domain, and VP2, which contributes to the viral capsid and is involved in virus-receptor binding [13,14]. The VP1 and VP2 regions are of particular interest, with up to 3% variability at the amino acid level [15]. Mutations in these genes can impact the virus’ ability to replicate, escape immune detection, or alter its interaction with the host.

Based on the phylogenetic analysis of the NS1-VP1u region, hB19V was classified into three genotypes: genotype 1, genotype 2, and genotype 3 [16,17]. Genotype 1 is prevalent worldwide and can be divided into two subgroups (genotype 1a and 1b); genotype 2 is commonly harbored in tissues of elderly people, but it is only sporadically detected as a circulating virus; while genotype 3 is only found in restricted areas, such as western Africa [18,19]. This last genotype shows a wider genetic variability in comparison with the other genotypes, and for this reason, it may show a more limited geographical distribution.

This research examines the surge of hB19V cases from January 2023 to September 2024, in the Siena area, Tuscany, with the aim to present an overview of genotypic characterization of hB19V positive cases in this area.

## 2. Materials and Methods

### 2.1. Clinical Samples

During the 2023–2024 period, 550 samples were processed for presumptive infection with hB19V at the Microbiology and Virology Unit of ‘S. Maria delle Scotte’ Hospital in Siena (Italy).

Among all the samples, 40 out of 181 blood samples were positive for hB19V by molecular assay, and 59 out of 369 serum samples were anti-hB19V IgM and IgG positive by serological investigation. The detection of both IgM and IgG was indicative of acute hB19V infection. Blood and serum samples of the same patient were only available for 24 subjects. Ethical approval was obtained from the local Ethical Committee for clinical trials (Protocol BIOBANCA VIROMICRO-2023, approval no. 25836, 15 January 2024) (Comitato Etico Regionale per la Sperimentazione Clinica della Toscana—sezione AREA VASTA SUD EST) in terms of General Data Protection and Regulation (GDPR) upon written informed consent signed by all subjects prior to participating in this study.

### 2.2. Serological and Molecular Analysis

The nucleic acid extraction was performed with the automatic extractor EZ1 and the DNA Blood kit (Qiagen GmbH, Hamburg Germany). HB19V viral load of blood samples was detected performing real-time-PCR using the Parvovirus B19 R-Gene^TM^ kit (Biomerieux, Florence, Italy), according to the manufacturer’s instructions. The exact sequences of the primers used in the kit are typically not disclosed publicly, however, they are carefully selected to match highly conserved regions of the NS1 or VP2 genes to ensure specificity and sensitivity. Serological IgM and IgG analysis of patient sera was carried out by using the LIAISON^TM^ Biotrin Parvovirus B19 IgM and IgG kit on the Liaison XL platform (Diasorin S.p.A.; Vercelli, Italy), according to the manufacturer’s instructions.

This test uses chemiluminescence immunoassay (CLIA) technology for the qualitative determination of specific IgG and IgM antibodies for Parvovirus B19. Results are reported in arbitrary units (U/mL). The manufacturer considers samples with values <0.9 U/mL as negative, >1.1 U/mL as positive, and between 0.9 and 1.1 U/mL as equivocal.

### 2.3. Next Generation Sequencing

Before starting the NGS protocol, the quality of extracted genetic material was assessed using a Nanodrop^TM^ spectrophotometer (Thermo Fisher Scientific, Waltham, MA, USA) by evaluating the A_260_/A_280_ ratio that provides information about sample contamination. The quantity of the total nucleic acid was then assessed with a Qubit DNA high-sensitivity assay kit and Qubit 3.0 fluorometer (Thermo Fisher Scientific, Waltham, MA, USA). Twenty-three samples were selected for NGS analysis. Library preparation was carried out following the Illumina RNA Prep with Enrichment kits (Illumina S.r.l. Milan, Italy) according to the manufacturer’s instructions. Briefly, 8.5 μL of extracted RNA, by the methodology described above, was denatured, followed by first- and second-strand DNA synthesis. This was followed by tagmentation, which uses enrichment bead-linked transposomes (BLT) to tagment double-stranded cDNA. This process fragments cDNA and adds adapter sequences. After tagmentation, the fragments were purified and amplified to add index adapter sequences for dual indexing. An index set, containing 96 unique, single-use Illumina DNA/RNA UD indexes, was used. Following clean up, 7.5 μL of the library was used for hybridization using oligos from the respiratory viral panel. This was followed by bead-based capture of hybridized probes, amplification, clean up, and quantification of the enriched library. Normalized libraries diluted to an equimolar concentration of 10 pM were then pooled and denatured according to Illumina’s instructions and charged on a V2 micro MiSeq 300 cycles flow cell (Illumina S.r.l. Milan, Italy). FastQ files were analyzed with the BaseSpace™ platform by Illumina with the Dragen targeted microbial app (Illumina S.r.l. Milan, Italy). All obtained sequences were uploaded on Genbank (https://www.ncbi.nlm.nih.gov/nucleotide/, accessed on 29 November 2024) with the following accession numbers: PQ660663; PQ660664; PQ660665; PQ660666; PQ660667; PQ660668, PQ660669; PQ660670; PQ660671; PQ660672; PQ660673; PQ660674; PQ660675; PQ660676; PQ660677; PQ660678; PQ660679; PQ660680; PQ660681; PQ660682; PQ660683; PQ660684; and PQ324621.1.

### 2.4. Phylogenetic Analysis

Sequences were aligned using the Geneious software (version 2025.0.2) with the MUSCLE algorithm, using the default parameters (three iterations and gap opening penalties). After calculation of pairwise distances to determine how similar or different each sequence is from the others. The phylogenetic tree was generated using the neighbor-joining algorithm using the Jukes–Cantor distance model, which assumes equal and constant mutation rates between all nucleotide pairs. Bootstrapping and reconstitution were carried out with 1000 replicates to obtain the confidence level of the phylogenetic tree. Sequence homology comparison was carried out with reference sequences representing the three main hB19V genotypes from the Gen Bank genetic sequence database: genotype1A: NC_00083.2; AF162273.1; genotype 1B: DQ357065.1; genotype 2: AY044266.2; AY064475.1; genotype 3: AY083234.1; and AX003421.1.

### 2.5. Evolutionary Rate and Selection Analysis

Selection pressure was analyzed in three proteins of hB19V based on the genome dataset.

All the sequences were aligned using the MUSCLE algorithm on the Geneious software. SLAC methods (www.datamonkey.org) were used to calculate ω values (ω = dN/dS) of B19V, where dN stands for the nonsynonymous substitution rate and the dS is for the synonymous substitution rate.

## 3. Results and Discussion

From the beginning of 2023 to September 2024, a total of 550 requests for hB19V diagnosis, both by serological and molecular methods, came to the attention of the Microbiology and Virology Unit at ‘S. Maria delle Scotte’ Hospital, based in Siena, Tuscany. Among them, ninety-nine samples were positive for hB19V (18%), of which, only three were collected in 2023 (two in April and one in October 2023).

**Figure 2:**
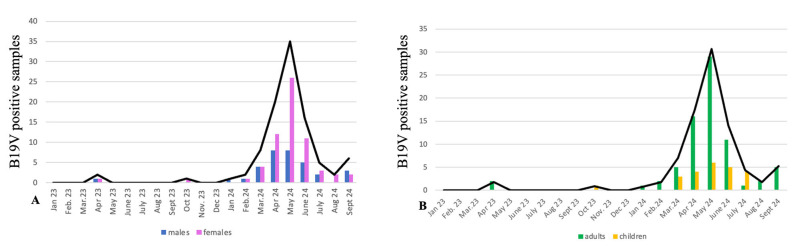
Parvovirus B19 cases from 2023 to the end of September 2024 in Siena area, Italy. (**A**) Number of positive samples distributed by gender; blue bars are from males and purple bars from females. (**B**) Number of positive samples distributed by age; green bars represent adult patients (>15 years old), and yellow bars correspond to children. This study showed that the hB19V infection was more frequent in adult females.

Our data show an increased number of positive samples since the first months of 2024 (Figure 2), reaching the highest peak in late spring/early summer 2024, according to the reported scientific literature [9]. A higher number of female subjects tested positive (63 out of 99), while the remaining 36 were male subjects. Twenty-four samples were from pediatric patients (0–14 years old), whereas the remaining seventy-five samples were from adults (>15 years old).

This increase in the number of hB19V-positive samples was partially attributed to the impact of COVID-19 restrictions both on viral circulation and immunity patterns. Studies and reports highlight that strict public health measures during the pandemic, such as social distancing, school closures, and reduced social interactions, significantly disrupted the transmission of various infectious diseases, including hB19V [20,21,22,23]. These measures led to a decline in population level immunity, particularly among children, who missed typical exposures to common pathogens [24,25]. Our study showed females were more affected than males. The higher prevalence in women might be attributed, in part, to their greater likelihood of caring for young children, potentially enhancing viral exposure. This may represent a problem, since non-immune pregnant women are at risk for fetal infections by hB19V, leading to greater complications, especially if the infection occurs in the first or second trimester. It has been widely documented that Parvovirus infection during pregnancy can be passed to the fetus in 30–50% of cases, with a high risk of developing anemia, fetal hydrops, and in some cases pre-birth death [26]. It is not known if pregnant women were present in our cohort, since clinical data were not available.

This high number of positive cases allowed us to characterize the circulating B19V genotype circulating in this outbreak.

As mentioned before, three different hB19V genotypes were defined: genotype 1, divided in two subgroups (1a and 1b), genotype 2, and genotype 3. Although genotype 1a is the most frequent worldwide, it appears to have been partially substituted by genotype 2 in the last 50 years; but nowadays, it is sporadically detected only in rare cases, and usually in elderly people. Genotype 3 is less frequent, and it is generally associated with peculiar, restricted areas [14,27].

**Figure 3:**
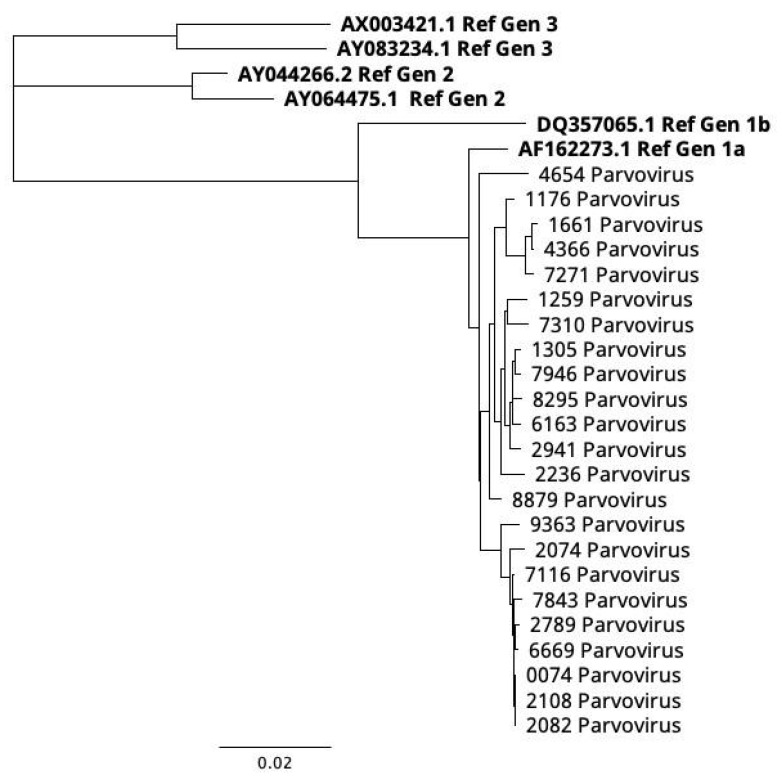
Phylogenetic tree of NGS samples. The phylogenetic tree shows that all samples were clustered under the genotype 1a. No samples were clustered under the genotype 1b, genotype 2, and genotype 3. The used references are highlighted in bold, all of them are accessible in the Genbank database. Genotype 1A: NC_00083.2; AF162273.1; genotype 1B:DQ357065.1; genotype 2: AY044266.2; AY064475.1; and genotype 3: AY083234.1; AX003421.1.

For this purpose, 23 samples with PCR-Ct between 25 and 36 were selected for a next generation sequencing (NGS) analysis. The following bioinformatic analysis with the Dragen Microbial Enrichment Plus software (ver 1.1.0) (Illumina^TM^) identified hB19V in 100% of sequenced samples. The mean coverage was 98.5% in all samples with a mean depth of 115X. The consensus sequences were generated and aligned with the CLUSTAL and MUSCLE algorithm with a minimum GC content of 50%. All aligned sequences were used to conduct a phylogenetic analysis; the three genotype references were taken from Genbank (gen1a: AF162273.1; gen 1b: DQ357065.1; gen2: AY064475.1; AY044266.2; gen3: AX003421.1; and AY083234.1). The phylogenetic tree was constructed based on the neighbor-joining method with 1,000 bootstrap repetitions (cutoff 50). The resulting phylogenetic tree (Figure 3) shows that all samples were clustered under genotype 1a and were closely related with each other (d = 0.008; d.s = 0.0062) with a distance of 0.147 against genotype 3, 0.128 against genotype 2, and 0.058 against genotype 1b; 0.2% of nucleotide divergence and 3% of amino acid divergence.

These results enhance the close relationship in the evolutionary history of all those isolates, showing few genetic differences.

All detected mutations were point mutations, and the analysis revealed a broad polymorphic distribution, with frequencies ranging from 13% to 100%. Of these, 40.4% (59/146) were located in the NS1 gene, 8.9% (13/146) were exclusive to the VP1 gene, and the remaining 50.7% (74/146) were in the VP2 gene, which shares its sequence with VP1, but differs by 227 amino acids (Supplementary Material Figure S1). These data (Table 1) demonstrate a greater variability in the VP1/VP2 genes according to the published literature [28]. The mutation rate per base length was calculated and estimated to be µ = 0.24 for the NS1 gene, µ = 0.34 for the VP1, and µ = 0.29 for the VP2.

In addition, the dN/dS ratio was calculated for NS1, VP1, and VP2 genes, obtaining for all of them a value which is less than 1: ω = 0.023 for NS1, ω = 0.040 for VP1, and ω = 0.050 for VP2 (dev st for: NS1 = 0.01, VP1 = 0.01; and Vp2 = 0.02), respectively.

The low ω values observed for the three examined proteins suggest a strong negative selection which can act to preserve the critical functions of the proteins which are vital for the virus life cycle. Similar results have been reported also in other studies [29,30,31].

Further studies are urgently needed to assess the distribution of these variants over time. The cohort must certainly be expanded to better understand the distribution and role of these variants in the long term. 

The translation of the sequences revealed the presence of non-synonymous mutations (listed in Table 2) within our cohort of samples. Among these changes, five were present in NS1 protein, three in VP1, and three in VP1/VP2. The mutation rates per base for the non-synonymous mutations in the proteins NS1, VP1, and VP2 were calculated to understand the evolutionary pressures acting on these viral components. The results show a significantly higher mutation rate for NS1 (0.3333 mutations per base) compared to VP1 (0.1265 mutations per base) and VP2 (0.0121 mutations per base). These findings suggest that while VP2 is under stronger negative selection to maintain its structural and functional integrity as a capsid protein, NS1 may tolerate more mutations, possibly due to its involvement in immune evasion [32]. VP1 shows an intermediate mutation rate, indicating a balance between evolutionary flexibility and the need to preserve viral function.

It has been documented that variations in the VP1/VP2 capsid proteins may influence the virus interaction with host immune responses and cellular receptors, potentially enhancing its ability to evade immunity and spread more efficiently within the population [32]. These mutations were randomly dispersed among the analyzed genomes and never present in all samples. However, while most of the changes occurred among amino acids similar for physicochemical properties, the replacement of positively charged lysine with negatively charged (K14E) aspartic acid in VP1u could affect the protein. This mutation could have significant consequences for the virus, including changes in capsid proteins, immune evasion, and infectivity. 

Indeed, this mutation is located in the N-terminal 1-80 aa of VP1u, which is rich in neutralizing epitopes [33]. In this regard, a deficient immune response to VP1u has been associated with persistent infections, emphasizing the important role of the immune response against VP1u in clearing the virus [34,35]. However, in this study, it was not possible to correlate this mutation with a more severe or prolonged disease, because samples were collected for diagnosis without knowing the subjects’ clinical status. All of the mutations found in hB19V isolates and summarized in Table 2 have already been reported in scientific literature, such as the F554S amino acid change, localized in the carboxy-terminal part of NS1 gene, and detected in 9/23 subjects [36]. This region, being highly polymorphic, was identified as exposed to positive selective pressure [30,31].

In conclusion, this is the first report on hB19V genotypes circulating during the 2024 outbreak in Tuscany, Italy. This recent surge in hB19V cases across different European countries, coupled with increased viral circulation and shifting epidemiological patterns, may signify a heightened exposure risk for a larger part of the population, including those traditionally considered at low risk, such as immunocompetent adults. Although often perceived as a benign childhood illness, this study reported an increase in infections, particularly in the adults. This underscores the need for a reassessment of risk stratification, even among immune competent adults and adolescents. Although the genotyping of the current circulating strain does not seem to show particular changes in hB19V genotypes, further studies, both on a larger court and on the protein conformation variability, could be needed to understand the possible impact of these mutations on proteins and their level of involvement in virus virulence and transmissibility.

Moreover, the recording and continuing observation of mutations, especially in limited areas, is important to inform health authorities and put in place recommendations and preventive measures. The use of existing non-specific surveillance systems and their adaptation to new health events is essential for effective epidemiological monitoring and control.

## Figures and Tables

**Table 1:** The nucleotide mutations shared by all samples analyzed with the NGS approach.

Gene	Mutations
NS1	803G>A; 1175 C>T; 1227 A>T, 1430 A>T; 1140 C>T; 1529A>G; 1928 A>C; 2286 T>C
VP1/VP2	2754 C>T; 3351T>G; 3444 T>A; 3489 C>T; 3531 A>C; 3752 C>G; 3795 A>G; 3894 C>T; 4264C>T; 43069A>T; 4345 T>C; 4346 C>A.

**Table 2:** List of amino acid mutations and their frequency in the hB19V sequences.

Gene	Mutation	Frequency
NS1	C17S	9/23
NS1	F57L	4/23
NS1	V71A	5/23
NS1	L111F	6/23
NS1	F554S	9/23
VP1u	K14E	7/23
VP1	V30L	18/23
VP1u	S98N	17/23
VP1/VP2	A260T-A33T	6/23
VP1/VP2	N533S-N306S	17/23
VP1/VP2	G537A-G310A	9/23

## Data Availability

Datasets analyzed or generated during the study can be provided, upon reasonable request, by contacting the corresponding author (mariagrazia.cusi@unisi.it).

## Supplementary Materials

The following supporting information can be downloaded at: https://www.mdpi.com/article/10.3390/pathogens14020121/s1, Figure S1: Illustration of the nucleotide variation frequencies detected in NS1 (a), VP1 (b) and VP2 (c) genes.
