# Supplementary material for: Genotypic Characterization of Human Parvovirus B19 Circulating in the 2024 Outbreak in Tuscany, Italy
Source: Pathogens. 2025 Jan 28;14(2):121. doi: 10.3390/pathogens14020121 (PMC11858505; doi:10.3390/pathogens14020121)
Supplement: Supplementary file 1 [file pathogens-14-00121-s001.zip › pathogens-3425433-supplementary.pdf]

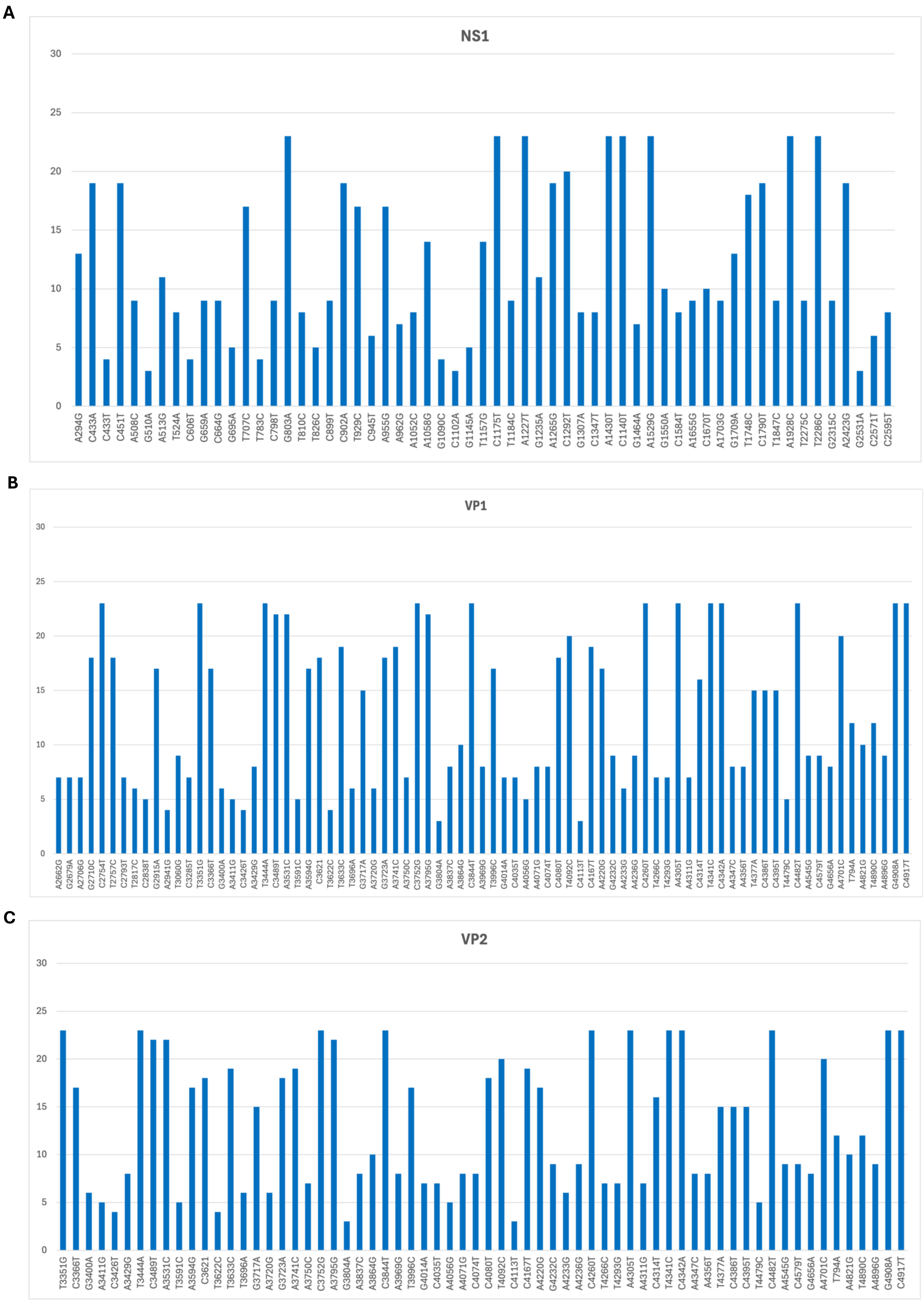
A
NS1
30
25
20
15
10
5
0
A294G C433A C433T C451T A508C G510A A513G T524A C606T G659A C664G G695A T707C T783C C798T G803A T810C T826C C899T C902A T929C C945T A955G A962G A1052C A1058G G1090C C1102A G1145A T1157G C1175T T1184C A1227T G1235A A1265G C1292T G1307A C1347T A1430T C1140T G1464A A1529G G1550A C1584T A1655G C1670T A1703G G1709A T1748C C1790T T1847C A1928C T2275C T2286C G2315C A2423G G2531A C2571T C2595T
B
VP1
30
25
20
15
10
5
0
A2662G G2679A A2706G G2710C C2754T T2757C C2793T T2817C C2838T G2915A A2941G T3060G C3285T T3351G C3366T G3400A A3411G C3426T A3429G T3444A C3489T A3531C T3591C A3594G C3621 T3622C T3633C T3696A G3717A A3720G G3723A A3741C A3750C C3752G A3795G G3804A A3837C A3864G C3844T A3969G T3996C G4014A C4035T A4056G A4071G C4074T C4080T T4092C C4113T C4167T A4220G G4232C A4233G A4236G C4260T T4266C T4293G A4305T A4311G C4314T T4341C C4342A A4347C A4356T T4377A C4386T C4395T T4479C C4482T A4545G C4579T G4656A A4701C T794A A4821G T4890C A4896G G4908A C4917T
C
VP2
30
25
20
15
10
5
0
T3351G C3366T G3400A A3411G C3426T A3429G T3444A C3489T A3531C T3591C A3594G C3621 T3622C T3633C T3696A G3717A A3720G G3723A A3741C A3750C C3752G A3795G G3804A A3837C A3864G C3844T A3969G T3996C G4014A C4035T A4056G A4071G C4074T C4080T T4092C C4113T C4167T A4220G G4232C A4233G A4236G C4260T T4266C T4293G A4305T A4311G C4314T T4341C C4342A A4347C A4356T T4377A C4386T C4395T T4479C C4482T A4545G C4579T G4656A A4701C T794A A4821G T4890C A4896G G4908A C4917T

Figure S1: Illustration of the nucleotides variation frequencies detected in (**A**) NS1 gene, VP1 (**B**) and VP2 (**C**).
